# Work-Related Musculoskeletal Symptoms in Surgeons Performing Total Knee Arthroplasty: An Exploratory Cross-Sectional Survey

**DOI:** 10.3390/jcm15135037

**Published:** 2026-06-28

**Authors:** Marina Sánchez-Robles, Carmelo Marín-Martínez, Joaquín Moya-Angeler, Vicente J. León-Muñoz, Francisco Lajara-Marco

**Affiliations:** 1 Department of Orthopaedic Surgery and Traumatology, Hospital General Universitario Rafael Méndez, Ctra. Nacional 340, Km. 589, 30817 Murcia, Spain; marina24sr@gmail.com; 2 Department of Orthopaedic Surgery and Traumatology, Hospital General Universitario Reina Sofía, Avda, Intendente Jorge Palacios 1, 30003 Murcia, Spain; camarin22@gmail.com (C.M.-M.); drlajaramarco@gmail.com (F.L.-M.); 3 Instituto de Cirugía Avanzada de la Rodilla (ICAR), C/Baritono Marcos Redondo 1, 30005 Murcia, Spain; jmoyaangeler@gmail.com; 4 Olympia Medical Centre, Paseo de la Castellana 259 E, 28046 Madrid, Spain; 5 Instituto Murciano de Investigación Biosanitaria Pascual Parrilla (IMIB), C/Campo 12, El Palmar, 30120 Murcia, Spain

**Keywords:** total knee arthroplasty, orthopaedic surgery, ergonomics, work-related musculoskeletal disorders, occupational health, surgeon health

## Abstract

**Background**: Work-related musculoskeletal disorders (WRMSDs) are highly prevalent among orthopaedic surgeons, but procedure-related symptom patterns, symptom intensity, and perceived interference remain incompletely characterised. This exploratory cross-sectional study evaluated the prevalence, anatomical distribution, and occupational consequences of musculoskeletal symptoms among surgeons performing total knee arthroplasty (TKA) and explored a non-validated descriptive discomfort–interference composite score. **Methods**: A cross-sectional survey was administered to a diverse group of 72 orthopaedic surgeons in Spain who performed TKA as part of their broader orthopaedic practice. Data on demographics, anatomical discomfort, global discomfort intensity, and functional interference were collected. A raw discomfort–interference score (DIIraw, 0–600) and a normalised score (DII100, 0–100) were calculated strictly as exploratory descriptors. Subgroup comparisons were performed according to the surgical position adopted during TKA. **Results**: The prevalence of musculoskeletal symptoms was 81.9%, with the lumbar spine (47.2%) and neck (38.9%) being the most affected regions. Mean global VAS discomfort was low (1.94 ± 2.08; median 1.0 [0.0–3.0]), although 15.3% of participants reported VAS ≥ 5. Self-reported physician-diagnosed work-related musculoskeletal conditions were reported by 51.4%, with 5.6% reporting sick leave and 6.9% reporting surgical intervention. DII100 values were low and skewed (mean 1.41 ± 2.31; median 0.25 [0.0–1.75]). No statistically significant differences were observed across surgical positions. **Conclusions**: Musculoskeletal symptoms were common among surgeons performing TKA, mainly affecting the lumbar and cervical regions, but average symptom intensity was low. A smaller subset reported clinically relevant discomfort and occupational consequences. The discomfort–interference composite score and any distribution-based values should be interpreted only as exploratory descriptors and require external validation. This study found no evidence of differences in musculoskeletal burden between surgical positions.

## 1. Introduction

Work-related musculoskeletal disorders (WRMSDs) represent a significant occupational hazard for surgeons, particularly within high-volume specialities such as orthopaedics [1,2]. Recent evidence suggests that the physical demands of adult reconstructive surgery (characterised by repetitive tasks, prolonged static postures, and significant physical exertion) place arthroplasty surgeons at a particularly high risk [2,3,4]. Specifically, female surgeons in this field have reported a high prevalence of repetitive musculoskeletal injuries, highlighting that individual and ergonomic factors may influence these outcomes [5]. Studies indicate that a substantial proportion of these professionals experience chronic pain, with some requiring practice modifications, medical leaves, or even early retirement due to these injuries [1,3,6].

In total knee arthroplasty (TKA), ergonomic strain is influenced by multiple factors, including operating table height, instrument design, and the surgeon’s positioning relative to the patient [4]. Recent simulations have demonstrated that optimising table height can significantly reduce postural load and perceived exertion during TKA [7]. Furthermore, while technological advancements, such as robotic assistance, have been shown to reduce physical stress and strain compared with conventional methods [8], the baseline musculoskeletal burden remains high across the speciality [9,10]. A recent systematic review focused on orthopaedic surgeons further supports this context, reporting a high prevalence of musculoskeletal (MSK) symptoms and injuries across orthopaedic procedures and emphasising the role of sustained non-neutral postures, repetitive high-magnitude forces, equipment, and operating-field height as procedure-specific biomechanical contributors [4].

Despite the known prevalence of these issues, existing instruments differ in the dimensions they capture. Region-based instruments such as the Nordic Musculoskeletal Questionnaire (NMQ) [11] provide a standardised framework for identifying the anatomical location of musculoskeletal symptoms and include items related to activity limitations, such as whether symptoms have prevented normal activities, including work, household duties, or leisure activities. However, this activity-limitation item is essentially dichotomous and does not quantify region-specific discomfort intensity or the frequency with which symptoms interfere with daily life or surgical activity. Thus, reporting that ankle discomfort has or has not prevented usual activities is not equivalent to quantifying a region-specific discomfort intensity during or after TKA and distinguishing whether the associated interference occurs occasionally, often, or daily.

Similarly, the Cornell Musculoskeletal Discomfort Questionnaire (CMDQ) incorporates frequency, discomfort, and interference dimensions and uses a multiplicative scoring logic to identify more severe musculoskeletal discomfort patterns [12,13]. However, it is not designed specifically for TKA. There is therefore a role for complementary, procedure-focused descriptive approaches that summarise anatomical burden, symptom intensity, and functional interference in the specific context of TKA. The present study was not intended to replace validated instruments such as the NMQ or CMDQ, but to explore a complementary, procedure-focused location–intensity–interference framework.

The purpose of this study was to evaluate the prevalence and anatomical distribution of musculoskeletal symptoms among surgeons performing TKA, to explore a non-validated discomfort–interference composite score as a hypothesis-generating descriptor, and to compare outcomes according to surgical position without inferring equivalence or superiority.

## 2. Materials and Methods

### 2.1. Study Design and Ethical Approval

A descriptive cross-sectional observational study was conducted using an anonymous, self-administered survey distributed to practising orthopaedic surgeons in Spain. The study protocol received approval from the Research Ethics Committee of Hospital General Universitario Reina Sofía, Murcia, Spain (protocol code CEI-HGURS/20–2022; approval date: 29 March 2022). Participation was voluntary and confidential. Completion of the questionnaire constituted informed consent for the processing of anonymous data.

### 2.2. Population, Sample, and Data Collection

The survey was distributed electronically via Google Forms (Google LLC, Mountain View, CA, USA) through professional networks and the Spanish Knee Society (SEROD), with two consecutive mailings conducted to optimise participation. In 2022, the SEROD mailing list comprised approximately 400 members. A total of 80 surgeons initially responded. Six respondents were excluded because they were residents or had fewer than three years of independent professional experience, and two responses were incomplete, leaving 72 valid complete questionnaires for analysis. This corresponds to an estimated dissemination response rate of 18% (72/400). Because the mailing list included surgeons with diverse orthopaedic practices rather than a closed registry of dedicated high-volume knee arthroplasty surgeons, this figure should be regarded as a dissemination response rate rather than the response rate of a closed registry of dedicated TKA surgeons. The response rate was slightly below the 20% benchmark reported in prior survey-methodology guidance cited in orthopaedic survey research [14,15], and this was addressed as a limitation. Inclusion criteria required current clinical practice in Spain, routine performance of TKA, and a minimum of three years of professional experience following specialist certification.

### 2.3. Measurement Instrument

The questionnaire was developed in accordance with general recommendations for designing questionnaires and interviews in clinical research, as described by Cummings and Hulley [16], and was specifically designed to assess musculoskeletal discomfort during or after total knee arthroplasty. Its structure incorporated three complementary dimensions of musculoskeletal burden: anatomical location, symptom intensity, and functional interference.

Although this region-based approach is conceptually related to instruments such as the NMQ, the full validated Spanish NMQ was not used because the survey had already been designed and distributed in 2022, before the publication of its Spanish cross-cultural adaptation and validation in 2024 [11]. In addition, the objective was to obtain a procedure-specific assessment of discomfort related to TKA rather than a general 7-day or 12-month prevalence measure. The NMQ includes an activity-limitation item, but it does not quantify region-specific discomfort intensity or graded interference frequency in the way explored in the present location–intensity–interference framework. Nevertheless, because the questionnaire was not a fully validated instrument, direct comparisons with NMQ-based studies should be interpreted cautiously. The English version of the procedure-specific questionnaire is provided as formal Supplementary Materials to support wider dissemination and future application in international contexts.

The questionnaire comprised four main sections:

- Sociodemographic and occupational aspects: age, sex, BMI, hand dominance, years of practice, professional setting, surgical subspecialty profile, and mean weekly TKA volume.
- Anatomical discomfort: discomfort was recorded across 12 body regions following the Corlett–Bishop scheme [17]. Global discomfort during or after TKA was recorded separately using a 0–10 visual analogue scale (VAS). For each anatomical region, participants selected interval categories of discomfort intensity (0, 1–2, 3–4, 5–6, 7–8, or 9–10). For aggregation into the regional score, these categories were coded as 0, 1, 2, 3, 4, and 5, respectively. The Regional Sum (SR) was calculated as the sum of the 12 regional scores (range 0–60). This categorical coding was used for the exploratory composite score and should not be interpreted as a validated transformation of the VAS.
- Functional interference: interference frequency was recorded in five categories (Never, Rarely, Sometimes, Often, Always) and coded as 0, 1.5, 3.5, 5, and 10, respectively, following the non-linear frequency weighting used in the Cornell Musculoskeletal Discomfort Questionnaire (CMDQ) [12,13,18,19]. In the CMDQ, frequency is not treated as a simple linear ordinal variable; instead, increasing weights are assigned to recurrent symptoms. In the present study, these weights were used as an exploratory burden descriptor rather than as a validated scoring system for TKA surgeons.
- Clinical and occupational consequences: self-reported physician-diagnosed musculoskeletal conditions, sick leave, previous surgery, symptom-related activity modification, and coping strategies during surgery were recorded. No independent verification of medical records was performed because the survey was anonymous and online.

### 2.4. The Discomfort–Interference Index (DII)

To provide an exploratory descriptive measure of overall anatomical discomfort and functional interference, two related discomfort–interference composite scores were calculated. The DII was not developed or validated as a psychometric instrument, diagnostic score, prognostic tool, or risk-stratification system. Throughout the manuscript, raw and normalised values are reported separately as DIIraw and DII100 and should be interpreted strictly as descriptive, hypothesis-generating metrics.

First, a raw score was calculated as DIIraw = SR × interference frequency score. SR ranges from 0 to 60, and the interference frequency score ranges from 0 to 10; thus, DIIraw ranges from 0 to 600. The multiplicative formulation was selected to reflect the burden-weighting logic used in the CMDQ, in which frequency, discomfort, and interference-related dimensions are multiplied to expand the score distribution and facilitate the identification of more severe combinations [12,13]. In this study, multiplication was also selected because it yields a score of zero when either anatomical burden or interference frequency is absent. This approach is conceptual and exploratory; alternative additive or weighted formulations were not assessed. Consequently, the DII should be regarded as a descriptive composite rather than a validated measurement scale.

Second, for interpretability, a normalised score was calculated as: DII100 = (SR/60) × (interference frequency score/10) × 100. This is equivalent to DII100 = DIIraw/6 and ranges from 0 to 100. In Excel terms, this corresponds to: = (SR_cell/60) × (Interference_cell/10) × 100.

The dataset-derived DII100 value exceeding 5 was retained exclusively as a descriptive indicator of the upper tail of the current distribution. Because this threshold was established within the same sample to which it was applied, it lacks established diagnostic or prognostic significance. Exploratory comparisons of sick leave and surgery indicated numerically higher DII100 values among affected participants; however, limited event counts and a lack of statistical significance were observed. Therefore, these findings should be regarded as hypothesis-generating only. Several methodological aspects of the DII formula require explicit clarification. First, the SR component may conflate anatomical breadth with per-region severity, as both a single severe regional complaint and multiple mild regional complaints can yield similar total scores. Second, as with any composite metric, various combinations of anatomical extent, discomfort severity, and interference frequency may theoretically produce comparable values. Highly discordant symptom profiles, such as severe discomfort with infrequent interference or mild discomfort with frequent interference, are expected to be uncommon in clinical settings because greater symptom intensity typically corresponds to more frequent interference. Third, the interference weights are intentionally non-linear, particularly for the Always category, to reflect the perspective that permanent interference imposes a substantially greater functional burden than occasional or frequent interference. In this dataset, no respondent selected Always; therefore, the highest interference-frequency weight did not affect the observed DII100 distribution. Future validation studies should evaluate these assumptions and compare alternative formulations, such as maximum regional score, additive models, or independently weighted components.

### 2.5. Subgroups and Statistical Analysis

Participants were assigned to four subgroups based on the surgical position employed when operating the knee contralateral to the dominant hand: Group A (contralateral side), Group B (ipsilateral side), Group C (between the patient’s legs), and Group D (facing the joint). Comparisons between subgroups were performed according to surgical position. The questionnaire allowed participants to select only one position. Each participant was assigned to the position most often used when operating the knee opposite their dominant hand. The survey did not capture mixed or proportional use of multiple positions.

Data analysis was conducted using IBM SPSS Statistics version 20.0 (IBM Corp., Armonk, NY, USA) and Python version 3.13.5 (Python Software Foundation, Wilmington, DE, USA). SPSS v20 facilitated data cleaning, descriptive statistics, and initial crosstabulations. Python 3.13.5 was employed to reproduce DII calculations, perform non-parametric and exact tests, conduct sensitivity analyses, and generate figures. Quantitative variables are reported as mean ± standard deviation or median [interquartile range] for skewed distributions. Comparisons across the four surgical positions used the Kruskal–Wallis test due to small, unbalanced subgroup sizes. Categorical variables were compared using chi-squared or Fisher’s exact tests, as appropriate. Statistical significance was defined as alpha = 0.05. No formal a priori sample size calculation was performed because the study was exploratory and descriptive; consequently, subgroup analyses were considered underpowered and were not interpreted as evidence of equivalence between positions. As a post hoc exploratory check, DII100 values and the dataset-derived DII100 > 5 value were compared according to self-reported physician-diagnosed conditions, sick leave, and surgery using Mann–Whitney U tests and Fisher’s exact tests. These analyses were not intended to validate the DII. An additional sensitivity analysis used the maximum regional score instead of SR as an alternative anatomical component, generating an exploratory DIImax100 score to assess whether subgroup findings depended on the use of a regional sum.

## 3. Results

### 3.1. General Characteristics of the Sample

A total of 72 valid complete questionnaires were analysed. The sample was predominantly male (73.6%), with a mean age of 45.1 ± 10.2 years and a mean BMI of 25.6 ± 3.4 kg/m^2^. Weekly total knee arthroplasty (TKA) volume varied: 44 surgeons (61.1%) performed fewer than two TKAs per week, while 28 (38.9%) performed two or more TKAs per week. This distribution indicates that the cohort represents surgeons performing TKA within heterogeneous orthopaedic practice rather than a closed population of dedicated high-volume arthroplasty surgeons. Specifically, 28 of 72 (38.9%) reported exclusive knee practice, 20 of 72 (27.8%) reported lower-limb practice including hip and knee surgery, and 24 of 72 (33.3%) worked in broader orthopaedics and traumatology, including general trauma, spine, shoulder, or other subspecialties. Additionally, 61 of 72 surgeons (84.7%) worked in public or mixed public–private settings (35 in public practice and 26 in mixed practice), where incomplete subspecialisation and general trauma responsibilities are common. Professional experience was substantial and varied, with a mean of 19.3 ± 9.9 years (range, 4 to 42 years). The distribution by experience strata is presented in Table 1.

### 3.2. Self-Reported Musculoskeletal Symptoms

**Figure 1:**
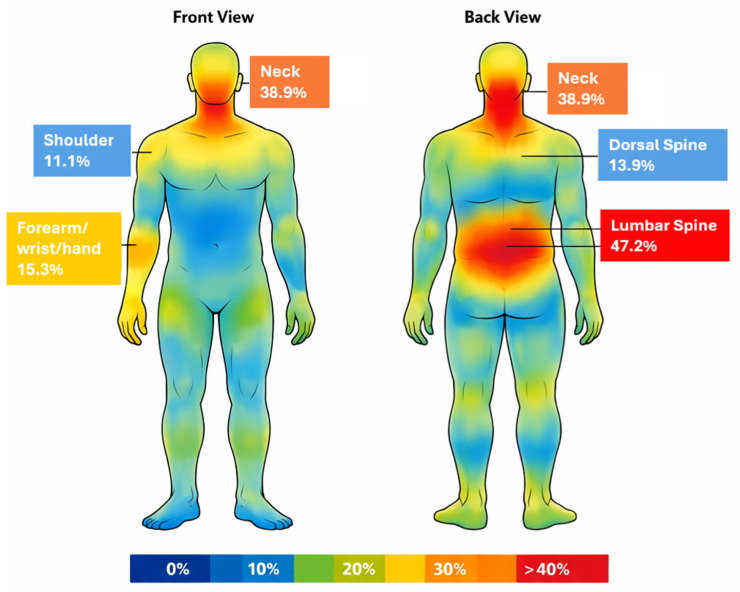
Regional prevalence of musculoskeletal symptoms. Colour-coded anatomical body map illustrating the distribution of musculoskeletal symptoms among orthopaedic surgeons performing total knee arthroplasty (n=72). Colour intensity represents symptom prevalence according to the scale shown, with warmer colours indicating higher prevalence. The percentages correspond to the proportion of surgeons reporting discomfort in each anatomical region.

Surgical-related musculoskeletal symptoms were reported by 81.9% of participants (59/72). As shown in the anatomical distribution (Figure 1), the highest prevalence was observed in the axial skeleton: the lumbar region (47.2%; 34/72) and the neck (38.9%; 28/72), followed by the forearm/wrist/hand (15.3%; 11/72) and the upper back (13.9%; 10/72). Exact regional counts and percentages are provided in Supplementary Table S1. The mean global VAS discomfort score after TKA was 1.94 ± 2.08, with a median of 1.0 [0.0–3.0], indicating generally low symptom intensity despite the high prevalence of any symptoms. Overall, 15.3% of the cohort reported VAS ≥ 5.

An exploratory descriptive assessment was conducted to evaluate lower-limb symptom lateralisation in relation to hand dominance. Among right-handed surgeons (n = 64), 7 (10.9%) reported right thigh or knee symptoms, and 9 (14.1%) reported left thigh or knee symptoms. Right leg or ankle symptoms were reported by 7 (10.9%), and left leg or ankle symptoms by 6 (9.4%). Among left-handed surgeons (n = 8), each of the four lateralised lower-limb regions was reported by 1 participant (12.5%). When symptoms were recoded relative to hand dominance, dominant-side lower-limb symptoms were reported by 14 of 72 participants (19.4%), and non-dominant-side symptoms by 15 of 72 (20.8%). Due to the small absolute counts and the possibility of bilateral symptom reporting, these data do not indicate a meaningful lateralisation pattern and should be interpreted descriptively.

### 3.3. Clinical and Occupational Consequences

The study identified the following self-reported clinical and occupational consequences among surgeons:

- Diagnosed Conditions: A total of 51.4% of respondents reported a self-reported physician-diagnosed musculoskeletal condition that they considered related to their work.
- Specific Diagnoses: The most common were low back pain/sciatica (29.2%), lateral epicondylitis (11.1%), plantar fasciitis (8.3%), and wrist/forearm tendinopathy (6.9%).
- Occupational Impact: 5.6% reported sick leave and 6.9% reported surgery due to these conditions.
- Coping Strategies: A total of 44.4% acknowledged ignoring symptoms, while 33.3% modified their posture. Only 1.4% reported adjusting theatre equipment.

### 3.4. Functional Impact and the Discomfort–Interference Index (DII)

Regarding interference with daily life, 23.6% of participants reported that symptoms affected them at least occasionally. DII100 values were low and skewed, with a mean of 1.41 ± 2.31 and a median of 0.25 [0.0–1.75] in the whole cohort. Among participants with non-zero DII values, the mean DII100 was 2.74 ± 2.60 and the median was 1.75 [1.0–4.08]. The dataset-derived value DII100 > 5 identified 5 participants in the upper tail of the observed distribution, corresponding to 6.9% of the total cohort and 13.5% of participants with non-zero DII values. This should not be interpreted as a validated clinical subgroup.

The distribution of interference-frequency categories was as follows: Never, 34/72 (47.2%); Rarely, 21/72 (29.2%); Sometimes, 15/72 (20.8%); Often, 2/72 (2.8%); and Always, 0/72 (0.0%). Consequently, no participant selected the highest frequency category, and the DII100 variance was not influenced by the top Cornell-derived weight of 10.

Exploratory criterion-related analyses indicated that DII100, as a continuous variable, was numerically higher among surgeons reporting sick leave (median 2.04 [1.31–4.23] vs. 0.13 [0.0–1.75]; *p* = 0.147) and surgery (median 1.75 [0.0–3.50] vs. 0.13 [0.0–1.69]; *p* = 0.344). However, these differences were not statistically significant, and event counts were limited. Similarly, participants with DII100 > 5 exhibited numerically higher rates of sick leave (20.0% vs. 4.5%; *p* = 0.255) and surgery (20.0% vs. 6.1%; *p* = 0.314), while the rate of self-reported physician-diagnosed pathology was comparable (60.0% vs. 50.7%; *p* = 1.000). As a result, no criterion validity or predictive performance can be established from these data.

**Figure 2:**
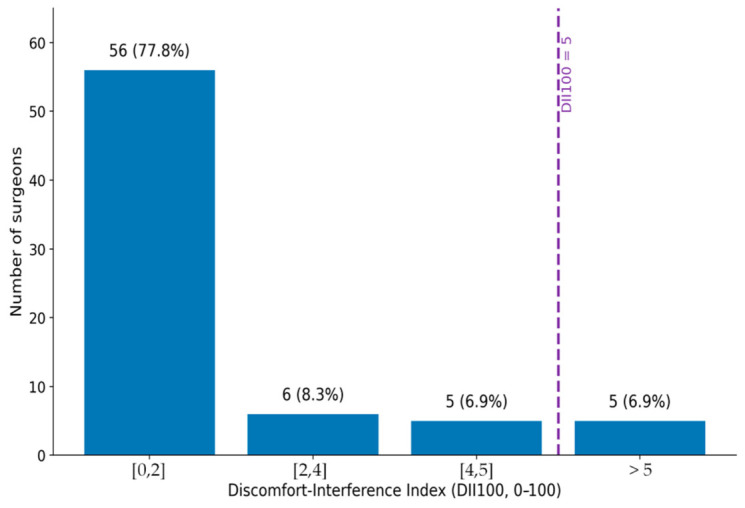
Distribution of the normalised Discomfort–Interference Index (DII100) scores among the study participants (n = 72). DII100 is calculated as (SR/60) × (interference frequency score/10) × 100 and theoretically ranges from 0 to 100. Values > 5 are shown only as a non-validated descriptive marker of the upper tail of the observed distribution. Bars show absolute frequencies with percentages in parentheses. The dashed vertical line represents the boundary at DII100 = 5.

In the post hoc maximum-regional-score sensitivity analysis, DIImax100 values were also low and skewed (mean 7.13 ± 9.55; median 3.00 [0.00–12.00]). These values demonstrated a strong correlation with DII100 (Spearman’s rho = 0.977; *p* < 0.001), and comparisons across surgical-position subgroups remained non-significant (Kruskal–Wallis *p* = 0.760) (Figure 2).

### 3.5. Subgroup Analysis by Surgical Position

Analysis of the four surgical positions used for contralateral TKA revealed no statistically significant differences in VAS, SR, DII100, or the number of affected regions (Table 2). Due to the small and unbalanced subgroup sizes, the lack of statistically significant differences cannot be considered evidence of equivalence between positions. Although descriptive values differed among groups, substantial within-group variability and considerable overlap in median and interquartile ranges were observed. None of the surgical positions demonstrated a consistent or statistically supported advantage across the evaluated outcomes. Therefore, the surgical-position analysis should be regarded as exploratory and hypothesis-generating. The maximum regional score sensitivity analysis similarly indicated no statistically significant differences among surgical positions.

## 4. Discussion

### 4.1. Prevalence and Anatomical Pattern

The results of this study confirmed that MSK symptoms are highly prevalent among orthopaedic surgeons performing total knee arthroplasty, with a prevalence of 81.9%. This high frequency aligns with recent literature reporting that work-related musculoskeletal disorders in orthopaedics range from 37% to 97% [1,2,3,11]. The most affected regions were the lumbar spine (47.2%) and neck (38.9%), consistent with the predominance of axial symptoms described in previous reviews of orthopaedic and surgical ergonomics [1,4,6]. However, symptom prevalence should be interpreted together with intensity: the mean VAS was low (1.94 ± 2.08; median 1.0 [0.0–3.0]), indicating that most reported symptoms were mild [20]. A smaller subgroup (15.3%) reported VAS ≥ 5, suggesting that clinically relevant discomfort was concentrated in a minority of respondents.

### 4.2. Clinical Outcomes and Occupational Impact

In addition to symptom prevalence, this study describes self-reported clinical and occupational consequences. More than half of participants (51.4%) reported a physician-diagnosed musculoskeletal condition that they considered work-related, with low back pain or sciatica (29.2%) and lateral epicondylitis (11.1%) being the most common. A smaller proportion reported sick leave (5.6%) or surgical intervention (6.9%). These findings suggest that musculoskeletal symptoms may have relevant occupational consequences for some surgeons, although the diagnoses and work-related attribution were self-reported and were not independently verified through medical records.

### 4.3. The Discomfort–Interference Index (DII): Exploratory Composite Descriptor

The DII was introduced as an exploratory composite descriptor to summarise the combined anatomical burden and frequency of functional interference. It should not be interpreted as a validated instrument, diagnostic scale, prognostic tool, or risk-stratification system. Traditional regional tools such as the Corlett–Bishop map and the NMQ characterise symptom location and prevalence, while the CMDQ combines frequency, discomfort, and interference dimensions in occupational settings [12,13,17]. In the present study, DIIraw and DII100 provided a simple descriptive summary of symptom burden in the context of TKA, but the formula was conceptual and has not undergone psychometric evaluation.

The dataset-derived value DII100 > 5 was retained only as a descriptive marker of the upper tail of the present distribution. Because it was derived from the same sample in which it was applied, it has no established diagnostic or prognostic meaning. The exploratory comparisons with sick leave and surgery showed numerically higher DII100 values among affected participants, but event counts were small and the differences were not statistically significant. These results should therefore be considered hypothesis-generating only.

### 4.4. Surgical Positioning and Professional Culture

Regarding surgical technique, no statistically significant differences were found between the four positions analysed. Therefore, this study provides no evidence that surgical position was associated with musculoskeletal burden, nor can it establish equivalence between positions because the subgroup sample sizes were small and unbalanced. Although a more frontal orientation to the operative field may theoretically reduce trunk rotation or asymmetric loading, this hypothesis was not confirmed by the present data and requires direct biomechanical assessment in larger, adequately powered studies.

Prior studies combining subjective and objective ergonomic measures have shown that surgeons frequently experience increased discomfort in the neck, upper back, and lower back during and after procedures, particularly when procedures are longer or involve demanding postures [21]. Observational studies using tools such as REBA have also demonstrated that poor cervical angle, standing posture, and lack of ergonomic setups contribute to ergonomic risk across surgical specialities [21,22]. Future TKA studies should combine self-reported symptom frameworks with objective methods such as wearable sensors, electromyography, RULA/REBA assessment, and intraoperative posture analysis to clarify the biomechanical mechanisms underlying surgeon discomfort.

This interpretation is consistent with previous surgical ergonomics literature. In a systematic review and meta-analysis of 40 studies including 5152 surgeons, Stucky et al. reported that work-related symptoms were highly prevalent among surgeons, with generalized pain in 68%, back pain in 50%, neck pain in 48%, arm/shoulder pain in 43%, and fatigue in 71%. Importantly, operating exacerbated pain in 61% of respondents, yet only 29% sought treatment for their symptoms [6]. The same review highlighted that limited awareness of ergonomic recommendations and absence of mandatory ergonomic training may delay the adoption of preventive strategies [6]. Park et al. similarly emphasised that surgeons may prioritise patient care while neglecting their own physical well-being [23]. Jensen et al. reported that ergonomics education during residency is often lacking and that structured educational sessions improved residents’ awareness of their own operating habits and their understanding of methods to prevent or treat work-related injuries [24]. Together, these findings support the need to move beyond individual tolerance and informal adaptation toward system-level interventions, including ergonomic training, operating-room layout optimisation, equipment adjustment, microbreak strategies, and formal incorporation of surgical ergonomics into orthopaedic training.

### 4.5. Limitations

This study has several limitations. First, the cross-sectional design precludes causal inference regarding the relationship between surgical position and musculoskeletal burden. Second, no formal a priori sample size calculation was conducted, as this exploratory, survey-based study aimed to describe musculoskeletal burden and generate hypotheses related to surgical position. The comparison of surgical positions involved small and unbalanced groups, particularly Group D, and was likely underpowered. Therefore, the absence of statistically significant differences should not be interpreted as evidence of equivalence or absence of any true ergonomic effect. Third, the estimated dissemination response rate was 18% (72/400), slightly below the 20% threshold referenced in prior survey-methodology guidance used in orthopaedic survey research [14,15]. Because respondents may have differed from non-respondents, self-selection and non-response bias may have affected prevalence estimates; surgeons with symptoms may have been more motivated to participate, whereas asymptomatic surgeons may have been less interested in the topic. Fourth, the SEROD mailing list included surgeons with diverse orthopaedic practices rather than a closed registry of dedicated high-volume TKA surgeons. The relatively low weekly TKA volume reported by many respondents, with 61.1% performing fewer than two TKAs per week, limits generalisability to high-volume arthroplasty surgeons and makes attribution of symptoms specifically to TKA uncertain. Nevertheless, this distribution reflects the actual organisation of orthopaedic practice in this setting, particularly in public and mixed public–private hospitals, where many surgeons perform knee arthroplasty alongside hip surgery, trauma, or other orthopaedic subspecialties. Fifth, although the survey followed a region-based approach comparable to established tools such as the NMQ, the full validated NMQ with its standard 12-month and 7-day time windows was not administered. Therefore, direct comparison with NMQ-based prevalence studies should be approached with caution. Sixth, the questionnaire and the DII are not validated psychometric instruments. The DII has not undergone formal assessment of internal consistency, test–retest reliability, construct validity, criterion validity, responsiveness, measurement error, or reproducibility. Similarly, the DII100 > 5 value is a dataset-derived descriptive marker and has no established clinical meaning. Seventh, symptoms, interference, diagnoses, sick leave, surgical treatment, and work-related attribution were self-reported and may be affected by recall and reporting bias; no medical record verification was performed. Finally, the low average VAS and low DII100 values indicate a floor effect in this cohort, which may limit the sensitivity of the composite score. Future research should validate the location–intensity–interference framework against external criteria such as work limitations, sick leave, treatment requirements, validated questionnaires, and objective ergonomic assessments, including wearable sensors, electromyography, REBA or RULA scoring, or intraoperative posture analysis.

Additional limitations pertain to the exploratory composite formula and the lateralisation analysis. The DII integrates anatomical spread with regional severity, and, as with any exploratory composite score, various combinations of anatomical extent, discomfort intensity, and interference frequency could produce similar values. Nevertheless, highly discordant profiles are considered clinically improbable, and assigning greater weight to the Always category is justified, as permanent interference imposes a substantially greater functional burden. Furthermore, no respondent selected Always in this sample, so the highest interference-frequency weight did not influence the observed results. The lateralisation assessment was included descriptively in response to observed right- and left-lower-limb variables; however, the small number of left-handed surgeons and the low absolute number of lower-limb symptoms limit the ability to draw meaningful conclusions about dominance-related symptom patterns.

## 5. Conclusions

In conclusion, work-related musculoskeletal symptoms were common among surgeons performing total knee arthroplasty, primarily affecting the lumbar and cervical regions. However, average symptom intensity was low, and clinically relevant discomfort appeared concentrated in a smaller subset. Self-reported physician-diagnosed conditions and occasional sick leave or surgery suggest that musculoskeletal symptoms may have occupational consequences for some surgeons. No statistically significant differences were observed between surgical positions, and the present data do not support ergonomic superiority or equivalence of any position. The discomfort–interference composite score should be viewed only as an exploratory descriptive framework; it requires formal validation before any clinical, diagnostic, prognostic, or risk-stratification use. The high rate of symptom neglect and the low use of equipment adjustment highlight the need for institutional ergonomic training, operating-room adjustments, and system-level prevention strategies to protect surgeon health.

## Figures and Tables

**Table 1:** Demographic and professional characteristics of the study population (n = 72).

Characteristic	Value
Age (years), Mean ± SD	45.1 ± 10.2
BMI (kg/m^2^), Mean ± SD	25.6 ± 3.4
Experience (years), Mean ± SD (range)	19.3 ± 9.9 (4–42)
Experience strata, % (n)	
<10 years	18.1% (13)
10–20 years	36.1% (26)
21–30 years	33.3% (24)
31–40 years	9.7% (7)
>40 years	2.8% (2)
Gender, % (n)	
Male	73.6% (53)
Female	26.4% (19)
Hand dominance, % (n)	
Right-handed	88.9% (64)
Left-handed	11.1% (8)
Main surgical practice, % (n)	
Exclusive knee practice	38.9% (28)
Lower-limb practice (hip and knee)	27.8% (20)
Other orthopaedic/trauma areas	33.3% (24)
Weekly TKA volume, % (n)	
<2 per week	61.1% (44)
2–4 per week	31.9% (23)
4–6 per week	4.2% (3)
6–8 per week	2.8% (2)
Clinical setting, % (n)	
Public	48.6% (35)
Mixed public–private	36.1% (26)
Private	13.9% (10)
Not reported	1.4% (1)
Surgical position, % (n)	
Group A (contralateral side)	27.8% (20)
Group B (ipsilateral side)	23.6% (17)
Group C (between legs)	30.6% (22)
Group D (facing joint)	18.1% (13)

**Table 2:** Comparison of clinical burden and the Discomfort–Interference Index (DII) according to surgical position. Values are presented as mean ± SD and median [IQR].

Variable	Statistic	Group A Contralateral (n = 20)	Group B Ipsilateral (n = 17)	Group C Between Legs (n = 22)	Group D Facing Joint (n = 13)	*p*
VAS (0–10)	Mean ± SD	2.35 ± 1.87	1.94 ± 2.49	1.86 ± 2.05	1.46 ± 1.94	0.425
	Median [IQR]	2.00 [1.00–3.25]	1.00 [0.00–3.00]	1.00 [0.00–3.00]	1.00 [0.00–2.00]	
SR (0–60)	Mean ± SD	4.20 ± 4.12	3.59 ± 3.37	4.86 ± 6.01	2.85 ± 2.91	0.827
	Median [IQR]	3.00 [1.00–6.00]	2.00 [1.00–6.00]	3.00 [1.00–6.75]	2.00 [1.00–3.00]	
DII100 (0–100)	Mean ± SD	1.60 ± 2.54	0.98 ± 1.79	1.68 ± 2.57	1.23 ± 2.23	0.690
	Median [IQR]	0.54 [0.00–2.19]	0.00 [0.00–1.50]	0.38 [0.00–2.12]	0.50 [0.00–1.25]	
Affected regions	Mean ± SD	2.05 ± 1.57	1.59 ± 1.28	2.14 ± 2.03	1.69 ± 1.32	0.796
	Median [IQR]	2.00 [1.00–3.00]	1.00 [1.00–2.00]	1.50 [1.00–3.00]	1.00 [1.00–2.00]	

## Data Availability

The data presented in this study are available on reasonable request from the corresponding author due to privacy and ethical restrictions.

## Supplementary Materials

The following supporting information can be downloaded at: https://www.mdpi.com/article/10.3390/jcm15135037/s1, Table S1: Exact regional prevalence of musculoskeletal symptoms among surgeons performing total knee arthroplasty (n = 72); Table S2: Lower-limb symptom lateralisation according to hand dominance (n = 72); Table S3: Distribution of interference-frequency categories used in the exploratory DII calculation.

## Abbreviations

The following abbreviations are used in this manuscript:

TKA	Total Knee Arthroplasty
WRMSD	Work-related Musculoskeletal Disorder
DII	Discomfort–Interference Index
SR	Regional Sum
VAS	Visual Analogue Scale
BMI	Body Mass Index
SEROD	Sociedad Española de Rodilla
