# Supplementary material for: Work-Related Musculoskeletal Symptoms in Surgeons Performing Total Knee Arthroplasty: An Exploratory Cross-Sectional Survey
Source: J Clin Med. 2026 Jun 28;15(13):5037. doi: 10.3390/jcm15135037 (PMC13362713; doi:10.3390/jcm15135037)
Supplement: Supplementary file 1 [file jcm-15-05037-s001.zip › jcm-4353494-supplementary.pdf]

Supporting information is provided as formal supplementary material accompanying the submission.

File S1 contains the English version of the surgical ergonomics and musculoskeletal burden survey, which is available for download at https://forms.gle/eS8524jY7CvzKFeq7 or by scanning the provided QR code.

Supplementary Table S1 details the exact regional prevalence of musculoskeletal symptoms. Supplementary Table S2 reports lower-limb symptom lateralisation according to hand dominance. Supplementary Table S3 summarises the distribution of interference-frequency categories.

Supplementary Table S1. Exact regional prevalence of musculoskeletal symptoms among surgeons performing total knee arthroplasty (n = 72).

**Body region**	**n/72**	%
Neck	28/72	38.9
Shoulders	8/72	11.1
Arm/elbow	5/72	6.9
Forearm/wrist/hand	11/72	15.3
Upper back	10/72	13.9
Middle back	7/72	9.7
Lower back	34/72	47.2
Buttocks/hips	7/72	9.7
Right thigh/knee	8/72	11.1
Left thigh/knee	10/72	13.9
Right leg/ankle	8/72	11.1
Left leg/ankle	7/72	9.7

Supplementary Table S2. Lower-limb symptom lateralisation according to hand dominance (n = 72).

**Hand dominance**	**n**	**Right thigh/knee**	**Left thigh/knee**	**Right leg/ankle**	**Left leg/ankle**	**Any dominant-side lower-limb symptom**	**Any non-dominant-side lower-limb symptom**
Right-handed	64	7/64 (10.9%)	9/64 (14.1%)	7/64 (10.9%)	6/64 (9.4%)	12/64 (18.8%)	13/64 (20.3%)
Left-handed	8	1/8 (12.5%)	1/8 (12.5%)	1/8 (12.5%)	1/8 (12.5%)	2/8 (25.0%)	2/8 (25.0%)
Overall	72	8/72 (11.1%)	10/72 (13.9%)	8/72 (11.1%)	7/72 (9.7%)	14/72 (19.4%)	15/72 (20.8%)

Note: right/left site variables and dominance-relative categories are not mutually exclusive because bilateral symptoms could be reported.

Supplementary Table S3. Distribution of interference-frequency categories used in the exploratory DII calculation.

**Interference category**	**Weight**	**n/72**	**%**
Never	0	34/72	47.2
Rarely	1.5	21/72	29.2
Sometimes	3.5	15/72	20.8
Often	5	2/72	2.8
Always	10	0/72	0.0

Note: no respondent selected Always; therefore, no DII100 variance was driven by the highest interference-frequency weight.
